# Buttocks Hard as Rocks: Not Wanted after Aortic Dissection Repair

**DOI:** 10.1055/s-0038-1639379

**Published:** 2018-07-27

**Authors:** Maude Cameron-Gagné, Luc Bédard, Valérie Lafrenière-Bessi, Marie-Hélène Lévesque, François Dagenais, Stéphan Langevin, Maxime Laflamme, Pierre Voisine, Frédéric Jacques

**Affiliations:** 1 Service of Cardiac Surgery, Institut universitaire de cardiologie et de pneumologie de Québec, Quebec City, Quebec, Canada; 2 Service of Orthopedics, CHU de Québec, Department of Surgery, Université Laval, Quebec City, Quebec, Canada; 3 Medical Imaging, Institut Universitaire de Cardiologie et de Pneumologie de Québec, Quebec City, v, Canada; 4 Intensive Care, Institut Universitaire de Cardiologie et de Pneumologie de Québec, Quebec city, Quebec, Canada

**Keywords:** gluteal compartment syndrome, type A aortic dissection repair, aortic dissection complication

## Abstract

The authors report the case of a patient developing a gluteal compartment syndrome after DeBakey type I dissection repair. Prompt recognition and treatment led to successful results. The surgical approach to the gluteal compartment is described.

## Introduction

A muscular compartment as hard as a rock is a cause for concern. The gluteal compartment or “buttock” is no exception. Gluteal compartment syndrome (GCS) is rarely reported. Failure to recognize this condition may lead to dramatic consequences. We report the case of a patient developing a GCS after type A aortic dissection repair. Prompt diagnosis and treatment led to a successful outcome. The surgical approach to the gluteal compartment is described. Permission to report this case was obtained from the institutional review board.

## Case Presentation

**Fig. 1:**
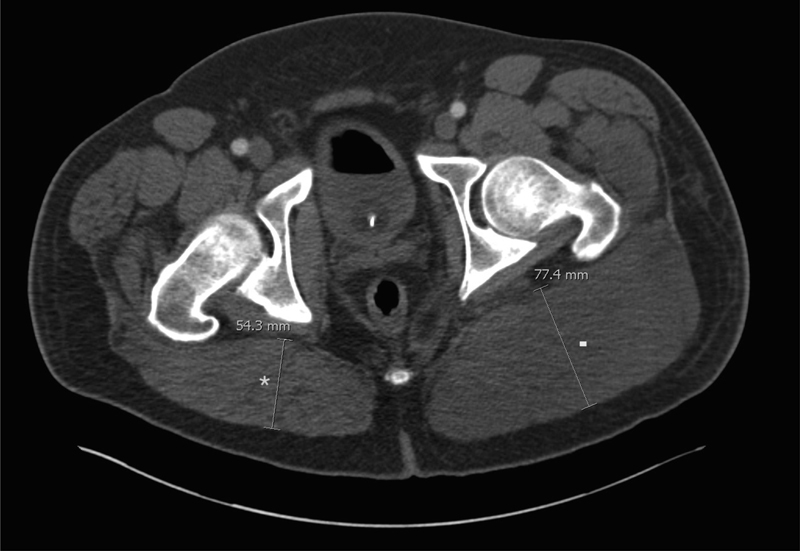
Computed tomography scan prefasciotomy showing net asymmetry of the gluteal muscles, especially the large left buttock measuring 77.4 mm (▪) versus 54.3 mm on the contralateral side (*).

**Fig. 2:**
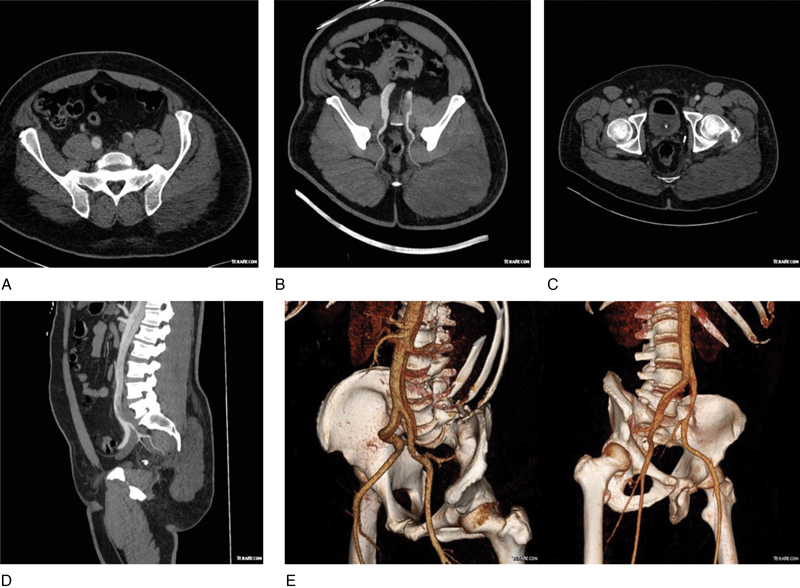
Computed tomography angiography prefasciotomy of maximum intensity projection. ( **A** – **D** ) and three-dimensional ( **E** ) reformatted images of the pelvis: ( **A** ) Axial: Dissection of bilateral common iliac arteries with thrombosed false lumen in the left common iliac artery; ( **B** ) Axial: Thrombosed false lumen in the left common iliac artery and patent left inferior gluteal artery; ( **C** ) Axial: Minimally decreased contrast opacification and minimally dilated left inferior gluteal artery compared with contralateral side; ( **D** ) Sagittal: Reformatted image showing dissection of the abdominal aorta extending in the left common iliac artery with poor opacification of the false lumen in the left common iliac artery. The false lumen is oriented toward the ostium of the left internal iliac artery, but the dissection is not extending into the left internal and external iliac arteries. The left internal iliac artery and its branches, the left superior and inferior gluteal arteries, are well opacified; ( **E** ) Dissection of the abdominal aorta with an intimal flap extending into bilateral common iliac artery. The false lumen is thrombosed within the left common iliac artery leading to a smaller size than the right. The left internal and external iliac arteries are well opacified.

A 41-year-old man, working in a furniture confection plant, experienced severe “tearing” chest pain upon heavy lifting. On presentation in another health facility, a chest computed tomography (CT) scan, with incomplete imaging of the iliofemoral axis, revealed a type A aortic dissection (DeBakey type I). Following transfer to our center, physical examination showed a pulseless left femoral artery, while no sign of acute limb ischemia was present. The patient underwent a mechanical Bentall procedure with hemiarch replacement under circulatory arrest for a total procedure time of 7 hours, 45 minutes. The immediate postoperative course was uneventful. The femoral pulses were symmetrical and well palpable once the patient rewarmed. Thirty-six hours after surgery, the patient was extubated and complained of severe pain to his left buttock. The lower limb pulses were present and no sensory or strength deficit was found. The left limb was normal, while the buttock was tense and painful especially upon flexion and adduction of the hip. A GCS was suspected. A CT angiography scan of the pelvis showed a dissection flap ending proximal to the iliac bifurcation without direct involvement of the iliac arteries. The false lumen was thrombosed and preferentially oriented toward the common left iliac artery. The opacification of the left internal and external iliac arteries was good. Preoperative CT angiography showed left gluteus muscle swelling ( Fig. 1 ). This was likely related to a malperfusion following thrombotic or embolic occlusion ( Fig. 2 ). The patient was diagnosed with GCS, further supported by a peak creatine kinase of 91 865 U/L.

**Fig. 3:**
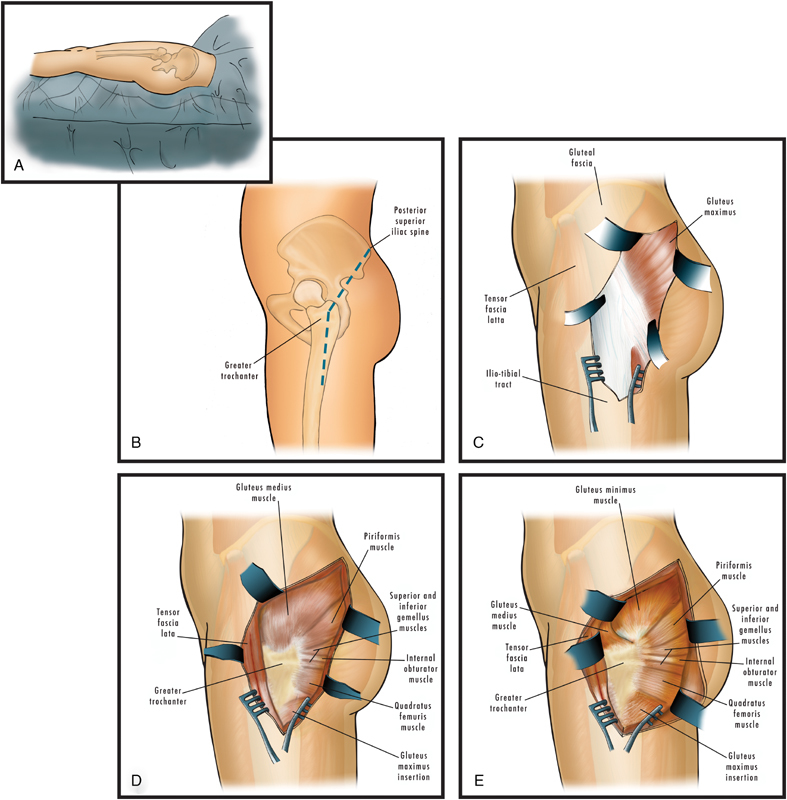
Kocher-Langenbeck approach: ( **A** ) Right lateral decubitus position; ( **B** ) Planned skin incision from the posterior superior iliac spine up to 5 cm of the greater trochanter and then to the lateral aspect of the femoral shaft; ( **C** ) Incision showing the gluteus maximus with fascia and iliotibial tract; ( **D** ) Gluteus maximus and iliotibial tract incision showing underlying structures with tensor fascia lata incised; ( **E** ) Anterior retraction of the gluteus medius muscle showing the gluteus minimus muscle.

A Kocher-Langenbeck procedure ( Fig. 3 ) was performed under general anesthesia with the patient placed in a right lateral decubitus position, with his legs bent at a 30° to 40° angle. An incision was made from the posterior superior iliac crest up to 5 cm of the greater trochanter and then to the lateral aspect of the femoral shaft. The tensor fascia latæ was fully opened longitudinally. The superficial and deep fascia of the gluteus maximus muscle and the fascia of the tractus iliotibialis were incised, revealing the heavily compressed muscles. The muscle bulged out of the incision. Muscles were then split along their fibers allowing opening of the fascia of the medius gluteus muscle where necrotic fibers were retracted anteriorly. The gluteus minimus muscle exhibited normal color. The left buttock was left open for 4 days, after which the skin was closed primarily, leaving the fascia open. Frequent dressing change was required as the edema was heavily seeping out of the wound. After 4 days, the muscle had significantly decreased in size and closing only the skin allowed the muscle to be completely tension free. The patient was discharged home on postoperative day 13. After 17 weeks, the patient returned to work and noticed intermittent claudication. Peripheral vascular impedance plethysmography and contrast-enhanced CT confirmed a 50% stenosis of the left iliac arterial axis. The patient was treated conservatively and is doing well without residual claudication 2 years thereafter.

## Discussion

Gluteal compartment syndrome is seldom reported. To date, one case has been reported before an endovascular abdominal aortic aneurysm repair 1 and four cases after acute abdominal aortic occlusion. 2 No case has been reported after thoracic aortic surgery.

The mechanism of GCS in our patient is most likely related to a temporary dynamic malperfusion of the left iliac artery axis in relation to an overpressurized false lumen. The reason why the gluteus maximus muscle was preferentially implicated is likely related to an isolated external iliac hypoperfusion brought by the dorsal decubitus positioning of the patient during the surgery. Regardless of the underlying mechanism, not recognizing this complication might have led to serious consequences such as myonecrosis, rhabdomyolysis, sepsis, severe acute renal insufficiency, multiorgan failure, and even death. 1

Diagnosis of GCS resides on a high index of suspicion since classic symptoms and signs, such as pain on passive movement, pallor, paresthesia, paralysis, poikilothermia, and pulselessness, are oftentimes not present in the buttock region. 3 In our patient, diagnosis was guided by the patient's symptoms of buttock pain. In addition, CT imaging depicted a thickened gluteal compartment.

Once identified, the GCS must be surgically addressed, in which case urgent decompression is the cornerstone of the treatment. The gluteal region is composed of the tensor fascia lata compartment and the gluteal compartments (maximus, medius, and minimus), all of which must be decompressed by fasciotomy (Fig. 3). 4 The buttock skin needs to remain open until edema resolves and primary closure without tension is allowed (4–10 days). If edema is still an issue on the 10th to 12th day, a skin graft may be necessary.

Herein, we report a case of GCS after type A aortic dissection repair. The patient was managed successfully with a fasciotomy of the left buttock. This complication may have been a result of a temporary occlusion of the left iliac axis with subsequent reperfusion of the femoral and gluteal regions. Prompt identification and surgical treatment of this syndrome led to a successful outcome.
